# Effect of immediate cold formalin fixation on phosphoprotein IHC tumor biomarker signal in liver tumors using a cold transport device

**DOI:** 10.1038/s41598-020-58257-3

**Published:** 2020-02-07

**Authors:** Melissa L. Lerch, Heidi L. Kenerson, David Chafin, Maria Westerhoff, Abbey Theiss, Michael Otter, Raymond S. Yeung, Geoffrey S. Baird

**Affiliations:** 1 Department of Laboratory Medicine, University of Washington Medical Center, Seattle, WA USA; 2 Department of Surgery, University of Washington Medical Center, Seattle, WA USA; 3 Ventana Medical Systems, Inc., 1910 Innovation Parkway, Tucson, AZ USA; 4 Department of Pathology, University of Michigan, 2800 Plymouth road, Ann Arbor, 48109 MIchigan USA

**Keywords:** Liver cancer, Tumour biomarkers, Diagnostic markers, Diagnostic markers

## Abstract

Phosphoproteins are the key indicators of signaling network pathway activation. Many disease treatment therapies are designed to inhibit these pathways and effective diagnostics are required to evaluate the efficacy of these treatments. Phosphoprotein IHC have been impractical for diagnostics due to inconsistent results occurring from technical limitations. We have designed and tested a novel cold transport device and rapid cold plus warm formalin fixation protocol using phosphoproteins IHC. We collected 50 liver tumors that were split into two experimental conditions: 2 + 2 rapid fixation (2 hours cold then 2 hour warm formalin) or 4 hour room-temperature formalin. We analyzed primary hepatocellular carcinoma (n = 10) and metastatic gastrointestinal tumors (n = 28) for phosphoprotein IHC markers pAKT, pERK, pSRC, pSTAT3, and pSMAD2 and compared them to slides obtained from the clinical blocks. Expression of pERK and pSRC, present in the metastatic colorectal carcinoma, were better preserved with the rapid processing protocol while pSTAT3 expression was detected in hepatocellular carcinoma. Differences in pSMAD2 expression were difficult to detect due to the ubiquitous nature of protein expression. There were only 3 cases expressing pAKT and all exhibited a dramatic loss of signal for the standard clinical workflow. The rapid cold preservation shows improvement in phosphoprotein preservation.

## Introduction

Cancer therapies often target specific proteins within pathways of biochemical networks. Precision medicine depends on the accurate diagnosis of disease using a variety of clinical assays to guide the selection of appropriate therapies^1^. Diagnostic assays must be performed on high quality tissue where the biochemical networks are preserved and representative of the biological state of the highly dynamic signaling network. One example of a precision medicine test is immunohistochemistry (IHC), such as the test for BRAF V600E mutation using the VE1 clone IHC used in colon, thyroid, and melanoma for prognostic value to guide treatment decisions^2,3^. BRAF V300E *assays*, like all IHC assays, are susceptible to preanalytical errors such as inadequate fixation or tissue processing, yet fixation and processing steps are often de-emphasized or taken for granted as an established part of the hospital workflow^1^.

Formalin fixation has been used for more than a century to preserve tissue for histopathology, our understanding of the biochemistry of formalin fixation is incomplete. There is evidence that cold formalin fixation improves the preservation of biochemical markers especially within signaling networks such as phosphoproteins^4,5^, and that cold formalin fixation has been shown to aid in the preservation of nucleic acids^6^. The rapid two-temperature (2 + 2) protocol was developed to take advantage of the biochemistry of formaldehyde in solution, where methylene glycol exists in equilibrium with formaldehyde in a temperature dependent manner^4^. At lower temperatures (4 °C), nonreactive methylene glycol is present in great excess over formaldehyde, and it and can effectively penetrate the tissue without reacting with tissue components. Also, at this lower temperature, enzyme kinetics slow and detrimental activity of degrading enzymes is likewise slowed. Raising the temperature (45 °C) then shifts the fixative’s equilibrium towards reactive formaldehyde, which actively crosslinks proteins and fixes the tissue. Recent technological advances have made monitoring fixation and tissue processing possible and could lead to standardization^7^.

Development of companion diagnostics alongside drug development can aid accurate diagnosis and treatment of disease^8^. Many signaling pathways are being targeted for drug development as promising drug candidates and phosphoproteins within those signaling pathways could be useful targets for diagnostics if technical challenges can be overcome. The PI3K-AKT pathway has been extensively targeted with nearly 40 candidates in clinical trials^9,10^. The utility of phosphoproteins in clinical trials has been limited by the lability of the phosphoprotein network. Cold ischemia time, the time a tissue specimen sits *ex vivo* prior to fixation, is another preanalytical variable that has a demonstrated and profound effect on measurements of signaling proteins like phosphoproteins^11–14^. There is a clinical imperative to study and develop approaches that control and monitor the temperature and time that specimens experience prior to fixation. We and others have found, for example, that rapid placement of tissues into cold formalin fixatives ameliorates some of the negative effects of prolonged cold ischemia time on measured levels of phosphoproteins, especially in larger tissue specimens that require longer fixation times^4–6,15^.

We designed an approach to improve the quality of surgically-excised tissue using a previously described cold transport device^16^ to facilitate the rapid collection, fixation, and monitoring of sensitive specimens for evaluation^17^. In this study we examined if the rapid cold condition could improve phosphoprotein IHC in liver tumors. Tumor tissue was split into two experimental conditions, a rapid cold fixation (aka 2 + 2, 2 hr cold + 2 hrs warm) and room-temperature fixation of the same duration, which was compared to tissue collected by clinical staff according to the current standard of care (including variable cold ischemic time followed by variable room temperature formalin fixation, generally overnight). We focused our analysis on the hepatocellular carcinomas and metastatic gastrointestinal carcinomas to the liver using phosphoprotein IHC biomarker analysis. We evaluated the preservation of five phosphoproteins: pAKT1, pERK1, pSRC, pSMAD2, and pSTAT3 in all three conditions.

## Results

We collected tissue from 50 liver tumors over the course of one year from patients with liver tumors greater than 3 cm. The tumors in this study were generally resected for curative intent or debulking, and hence extensive diagnostic assessments were not clinically necessary. Tissue was excluded from the study in 10 cases (20%), when the patient’s tumor was not malignant (n = 3), there was no tumor present in the research tissue sample (n = 1), only one of the two tissue samples collected in the surgical suite contained carcinoma (n = 4), a post-fixation tissue processing error occurred (n = 1), the clinical tissue was not available due to incomplete consent (n = 1), and the research tissue sample was too small to meet our criteria for analysis (n = 1).

**Figure 1:**
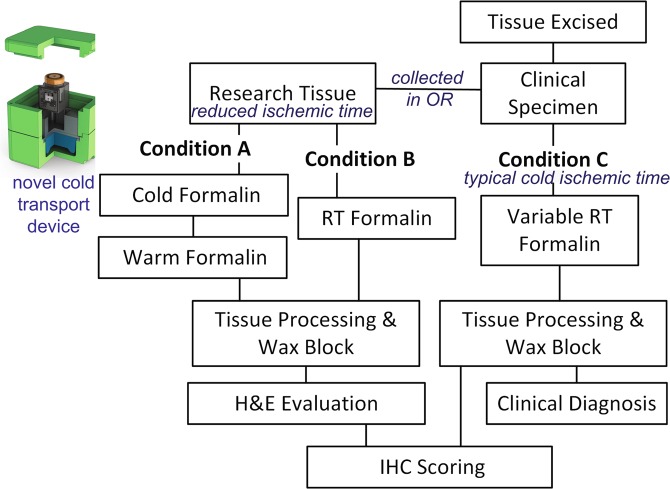
Experimental design for tissue collection and processing including diagram of cold transport device used in this study to improve phosphoprotein IHC.

We focused on the gastrointestinal metastatic lesions in the liver (n = 18, GI) and the hepatocellular carcinoma tumors (n = 10, HCC) to determine if our cold transport system and rapid processing protocol could improve phosphoprotein IHC. Tissue was collected directly in the operating room by placing resected material into either cold formalin (Condition A) or room temperature formalin (Condition B). Temperature was maintained in Condition A by transporting tissue within the cold transport device with a custom data logger that records the time of fixation, temperature, and transport specific parameters (including leaked fixative or aberrant acceleration, i.e. “dropping” the specimen, Fig. 1). Tissue in Condition B was fixed for 4 hours at room temperature and thereafter processed under identical conditions as Condition A (Fig. 1). Blocks were obtained from Pathology to use as a control for the routine clinical workflow. The experimental tissue was from adjacent tumor sections while the physical relationship to the clinical tumor is unknown in most cases.

### Phosphoprotein scoring

**Figure 2:**
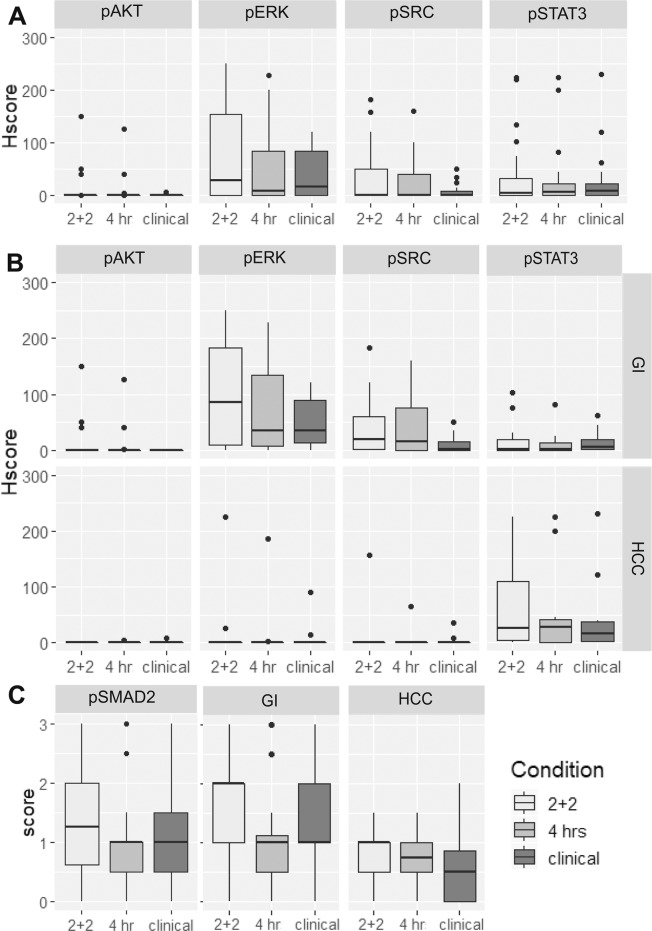
Phosphoprotein IHC scoring. (**A**) Combined hepatocellular (HCC) and metastatic gastrointestinal carcinoma (GI) liver tumors. (**B**) IHC scores by Phenotype: GI (top panel) and HCC (bottom panel). (**C**) pSMAD2 scoring by phenotype.

**Figure 3:**
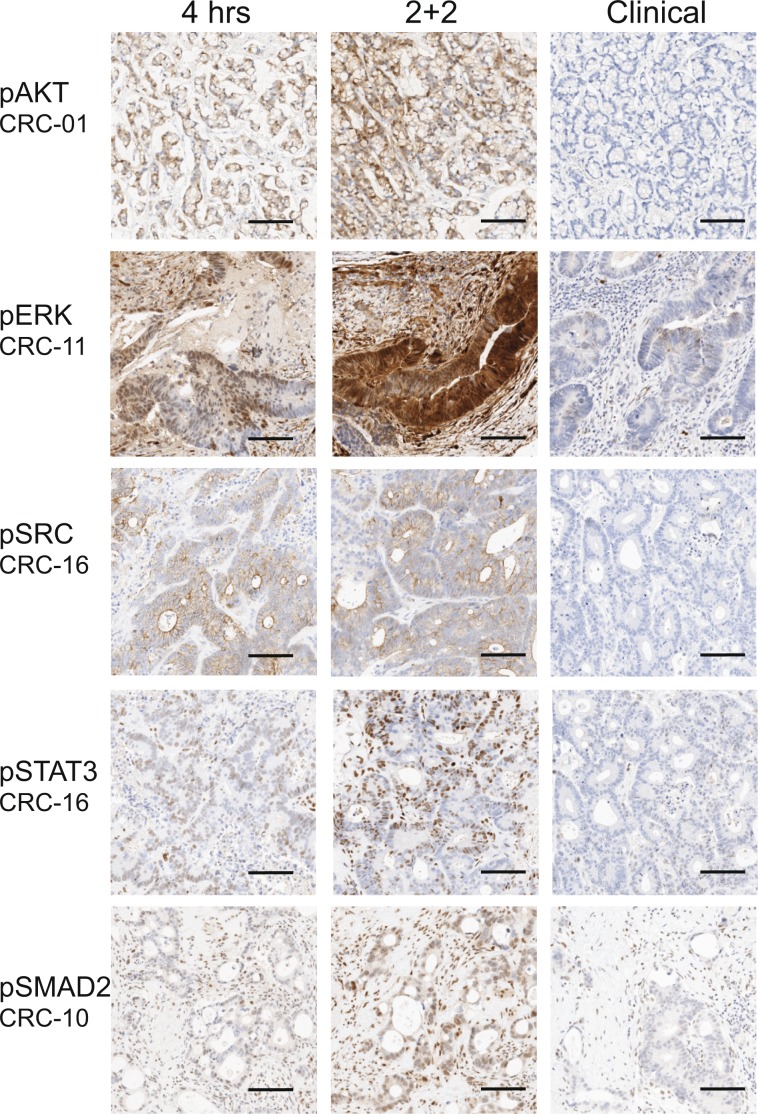
Representative IHC phosphoprotein images. (**A**) pAKT. (**B**) pERK. (**C**) pSRC. (**D**) pSTAT3. (**E**) pSMAD2. The scale bar is 100 µm.

Five phosphoproteins markers pAKT, PERK, pSRC, pSTAT3, and pSMAD2 were assessed by IHC (Table 1, Fig. 2). Representative images with positive-IHC for each phosphoprotein are shown in Fig. 3. The two experimental conditions were compared using two-sided Wilcox Signed Rank test. Three markers pERK, pSTAT3, and pSMAD2 showed statistically significant difference between 2 + 2 and 4 hr formalin fixation. P-values were then converted into false discovery rate q-values to correct for the multiple comparisons. Following this analysis only pERK remained statistically significant when compared to the clinical control tissue.

**Table 1:** Comparison of Pathologist H-scores between conditions for phosphor-biomarkers.

IHC	All cases			Wilcox Test			
	Median Score (25–75%ile)			2 + 2 vs 4 hrs		2 + 2 vs clinical	
	4 hrs	2 + 2	Clinical	p score	q-score	p score	q-score
pERK	8 (0–85)	25 (0–160)	20 (0–86)	0.0068	0.034	0.042	0.105
pSRC	0 (0–41)	1 (0–51)	0 (0–12)	0.167	0.178	0.031	0.105
pSTAT3	6 (0–24)	7 (0–34)	9 (1–24)	0.048	0.081	0.330	0.352
pAKT	0 (0-0)	0 (0-0)	0 (0-0)	0.178	0.178	0.093	0.154
pSMAD2*	1 (0.5–1)	1.5 (0.5–2)	1 (0.5–1.875)	0.025	0.063	0.352	0.352

pSMAD2 was scored on a simple 0, +1, +2, or +3 scale for overall intensity because staining was ubiquitous but intensity varied (Table 1). There was not a significant difference between the two experimental conditions.

### Phenotypes

We analyzed the dataset by phenotype: metastatic GI tumors and primary hepatocellular tumors (Table 2, Fig. 2b). In the metastatic GI tumors (aka metastatic colorectal carcinoma (CRC)), pERK and pSRC expression was most common, while pSTAT3 or pAKT were infrequently expressed. In the hepatocellular carcinoma cases pSTAT3 was most frequently expressed with the exception of HCC-5 that exhibited strong expression for pERK, pSRC, and pSTAT3.

**Table 2:** Comparison of Tumor Phenotype for all phosphor-biomarkers.

IHC	CRC: Median score (25–75%ile)			HCC: Median score (25–75%ile)		
	4 hrs	2 + 2	Clinical	4 hrs	2 + 2	Clinical
pERK	30 (5–143)	61 (7–189)	34 (12–92)	0 (0–1)	0 (0–12)	0 (0–8)
pSrc	15 (0–75)	10 (0–60)	2.5 (0–23)	0 (0-0)	0 (0-0)	0 (0–2)
pSTAT3	2 (0–13)	3 (0–22)	6 (1–20)	28 (0–84)	26 (2–156)	16 (0–60)
pAKT	0 (0-0)	0 (0–21)	0 (0-0)	0 (0-0)	0 (0-0)	0 (0-0)
pSMAD2*	1 (0.5–1.25)	2 (1,2)	1.25 (1,2)	0.75 (0.375–1)	1 (0.375–1.125)	0.5 (0-1)

*pSMAD2 is scored on simple scale and not the H-score.

### AKT-positive metastatic GI tumors

**Figure 4:**
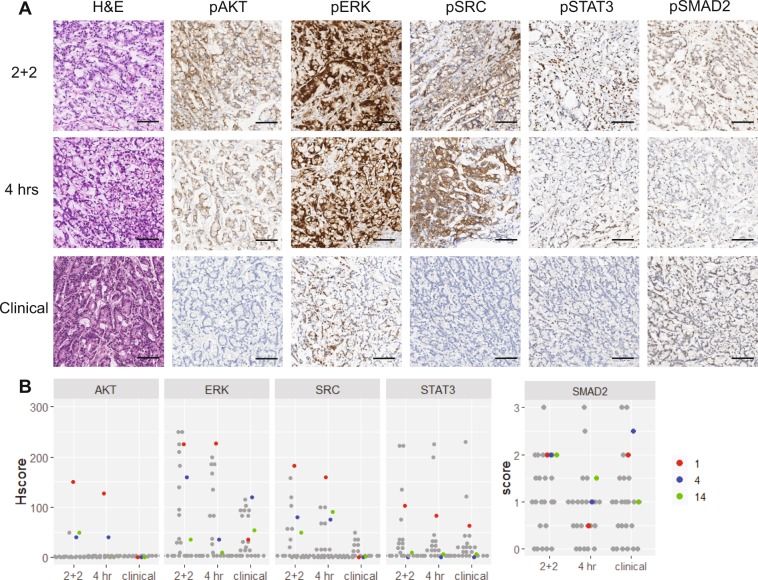
pAKT-positive GI phosphoprotein scores and representative IHC highlighting the loss of pAKT in routine clinical workflow condition. (**A**) Representative IHC for H&E, pAKT, pERK, PSRC, pSTAT3, and pSMAD2 for all three experimental conditions. (**B**) Dot plot of all IHC scores with the AKT-positive cases highlighted: CRC-1 (red), CRC-4 (blue), and CRC-14 (green). The scale bar is 100 µm.

There were only three cases exhibiting pAKT staining (Fig. 4). In all three cases there was a striking difference between the experimental and clinical conditions. Most notably pAKT and pSRC staining was present in the two experimental conditions and absent in the clinical control specimen accompanied by a marked decrease in pSTAT3 expression (Fig. 4).

## Discussion

In this study we demonstrate the use of a cold collection transport device coupled with rapid cold-hot formalin fixation and a standard processing protocol to generate high-quality tissue specimens for rapid turn-around of sensitive phosphoprotein biomarkers. We found that the novel device, paired with our previously published and validated fixation conditions, resulted in high quality tissue that in some cases yielded improved phosphoprotein preservation.

We analyzed five phosphoproteins under two experimental conditions: rapid cold-warm fixation and room-temperature fixation, and compared to the tissue from routine hospital workflows. We observe improvements in preservation of phosphoproteins using a rapid cold transport and preservation approach reducing ischemic time and inhibiting enzymatic activity that typically occurs at room temperature. Phosphoprotein stability will vary from protein to protein, and even patient to patient, due to kinase and phosphatase activity within the complex biological environment. Each target and antibody requires optimization for every disease as the enzymes present in liver tumors will differ from those present in breast tumors. The liver tumors in this study were large enough to allow for ample collection for diagnostic and research projects. However, we implemented strict criteria that all tissue samples had to meet the 4 mm minimum size for analysis similar to standard tissue grossing procedures. Smaller tissue pieces allow for rapid penetration of fixative and would not be representative.

In this study, two pathologists independently scored the slides and used semiquantitative H-scores for comparisons between experimental conditions and the clinical slides. H-scores provide a weighted evaluation of the intensity of the antibody signal in morphologically specific cellular components with the percentage of staining in tumor tissue. Adjacent normal tissue and nonspecific background staining was not scored, and hence any observed enhancements in staining were morphologically specific to the target compartments (nucleus, cytoplasm, membrane, etc). In general, enhanced staining resulting from the 2 + 2 protocol was not accompanied by increased off-target or background staining, consistent with an interpretation that the enhancement of immunoreactivity was specific to the intended target.

Tumor heterogeneity is a known complication in assessing tumor responsiveness to drugs^18^. In our study, we obtained one piece of tissue that was split into two experimental tissue fixation conditions that were located in an adjacent area of the tumor. The clinical control tissue was obtained from blocks from the Pathology Department of the hospital with an unknown physical relationship to the experimental tissue. In two comparisons between 2 + 2 cold-warm fixation condition and 4 hr fixation condition, pSTAT3 and pSMAD2, the p-score is less than 0.05 but increases above that threshold when the multiple comparison analysis is applied. Similarly, the 2 + 2 condition compared to the clinical handling condition pSRC has a p-value of 0.03 that increases to q-value of 0.105 following the multiple comparison correction. Only pERK 2 + 2 condition compared to 4 hr condition retains a q-value of 0.03 after the multiple comparison correction. It is possible that the control tissue complicates this comparison due in part to tumor heterogeneity. The tumors studies were all greater than 3 cm and the spatial difference could result in greater tumor heterogeneity between clinical and experimental conditions.

While ideally, every diagnostic test would be optimized for all preanalytical conditions, ideal fixation conditions for all tissue assays have not yet been established, and the lack of current fixation monitoring technology means that even if optimal tissue fixation conditions are established for specific assays, it may be difficult to ensure that every single clinical specimen receives this optimal treatment. In the practice of medicine today, the preanalytical procedures in most clinical assays are constrained by hospital workflows, and results vary accordingly. Promisingly, though, recent technological advances have made monitoring fixation and tissue processing possible, and these advances could lead to standardization of preanalytical processes^7^. The 2 + 2 rapid fixation protocol utilized here is one such standardized method, and it has been shown to perform well in a broad range of tissues and is amenable to adaptation within a monitoring system.

The cold transport device is easy to use, appropriate for the surgical suite, and when coupled with the rapid fixation protocol reduces the turnaround time of diagnostic testing. The reduction in ischemic time, combined with the cold formalin step, allows fixative to penetrate into tissue while simultaneously reducing degrading enzymatic activity, with the overall effect of improving phosphoprotein biomarker assessment. We have previously developed a time-of-flight ultrasound instrument to perform real-time monitoring of tissue fixation and processing. When the 2 + 2 protocol, the cold transport device, and the fixation monitoring device are combined, they form a comprehensive clinical quality assurance system that allows for the tracking, monitoring, and production of high-quality tissue to aid in the rapid generation of high quality diagnostic results.

This cold transport device has the potential to reduce the preanalytical variability observed in preservation and transport issues (25) by monitoring tissue temperature and time of fixation. When coupled with a radio frequency identification system (RFID) identifying both the specimen and transport box^19^, it adds an additional layer of assurance that the specimen is properly labeled and tracked to ensure proper identification of specimens. Monitoring fixation time could lead to better standardization of processing and lead to overall reduction of errors by improving reproducibility for higher quality tissue. Preanalytical monitoring enables laboratories to demonstrate and document regulatory compliance as it expands beyond breast tumor markers such as HER2, ER, and PR^11^ and into other disease biomarkers^20^ and enable the biorepository to better curate their collection of biospecimens for research purposes.

Our cold transport device is envisioned as part of a larger quality assurance program, when coupled with other preanalytical innovations using RFID tracking devices, rapid cold-hot fixation protocols, real-time fixation monitoring, and other innovations that ensure valuable tumor tissues can be optimally evaluated. These innovations could especially aid in the assurance that observed tumor heterogeneity is not an artifact from tissue handling discrepancies, confirming diagnosis for primary-metastatic lesion differences, and resolving biopsy-resection discordance in sensitive, critical diagnosis.

## Materials and Methods

### Data logger and cold storage device

The cold transport device consists of a foam-insulated box (CoolBox, Biocision) with a metal sample holder designed to fit a data logger that holds a sample collection vial (Fig. 1). The temperature is maintained with a cooling core (pre-chilled at −20 °C) and assembled with the metal holder 20-minutes prior to collection. The metal sample holder, formalin, and data loggers were pre-chilled at 4 °C prior to assembly.

Data loggers have several sensors allowing collection of temperature, position, time, and other variables during transport (Fig. 1).

### Tissue collection

Approval was granted through University of Washington Medical Center (UWMC) Institutional Review Board (#31281). Tissue was procured through the UWMC Liver Tissue Repository and informed consent was obtained from all patients prior to surgical resection to harvest fresh tissue not needed for pathologic evaluation (Fig. 1, Conditions A & B). Separate informed consent was obtained through UWMC tissue repository service, NW Biotrust, for the clinical specimen remaining after clinical testing (Fig. 1, Condition C). The methods were carried out in accordance with IRB guidelines and regulations.

Fresh tissue was collected directly in the operating room where a small portion of resected tissue was used for this study. The research tissue was equally divided between two conditions A and B (minimum of 4-mm core biopsies) and placed directly into dry containers with a formalin dispenser built into the lid (Biopsafe, Axlab, Denmark) to minimize formalin exposure in the operating room. After the specimen was placed in the container and closed, pressing a button on the lid punctured a receptacle containing formalin and immersed the specimen in fixative. For Condition A, the formalin was cold and the data logger was activated to record cold formalin incubation time. Tissue for Condition B was placed into room-temperature (RT) formalin. Cold ischemic time was kept to an absolute minimum by performing tissue acquisition in the operating room immediately upon tumor resection. The clinical specimen was processed as per usual clinical workflows and obtained as cut slides on glass referred to as Condition C.

### Tissue processing

Tissue for Condition A was fixed for 2 hours in 4 °C formalin and then 2 hours in 45 °C formalin (2 + 2)^4^ and tissue in Condition B was fixed for 4 hours at RT. Tissue was processed on a commercial tissue processor, Lynx II (Electron Microscopy Services) equipped with two Peltier stations that can cool and heat reagents. A standard overnight processing protocol was used with a variable 70% ethanol hold (10 min-6 hrs), 2x 60-min 90% ethanol, 3x 60-min 100% ethanol, 2x 60-min xylene, 1x 90-min xylene (45 °C) and 60-min wax. Tissue was placed into paraffin blocks and sectioned onto glass slides (4 μm). Tissue from Condition C was processed in the hospital’s clinical pathology laboratory and sectioned onto glass slides (4 μm).

### Immunohistochemistry

Immunohistochemistry was performed on an automated VENTANA Discovery XT staining instrument according to the manufacturer’s recommendations. Five different phosphorylated antibodies were utilized in this study from Cell Signaling Technologies: Phospho-AKT (clone D9E, dilution 1:50, Ser473, #4060), Phospho-ERK (clone 20G11, dilution 1:400, Thr202/Tyr204, # 4376), Phospho-SRC (clone D49G4, dilution 1:200, Tyr416. #6943), Phospho-STAT3 (clone D3A7, dilution 1:30, Tyr705, #9145), and Phospho-SMAD2 (clone 138D4, dilution 1:80, Ser465/467, #3108). Slides were deparaffinized using EZPrep (Ventana Medical Systems) at 90 °C, antigen retrieval, and antibodies conditions followed package inserts. Slides were developed using OptiView DAB detection kit (Ventana Medical Systems) and counterstained with hematoxylin. Whole slide images were obtained using an Aperio slide scanning system.

### Slide scoring

Slides were reviewed by two pathologists (MW, GB). pSMAD2 IHC stain intensity was scored using a simple semiquantitative scale (0 (none), 1+ (weak), 2+ (moderate), 3+ (strong). Cases in which the two pathologists differed in assessment by more than one semiquantitative score (1+ vs. 3+) were reviewed over a multiheaded scope and a consensus score was reached. Gross discrepancies generally resulted from low percentage of tumor cells present within the section analyzed.

Semiquantitative H-scores were assigned to pAKT, pERK, pSRC, and pSTAT2 by multiplying the percentage of cells specifically expressing the desired protein by their intensity (0–3+) using the following formula: [1 × (% 1+ cells) + 2 × (% 2+ cells) + 3 × (% 3+ cells)]. The final H-scores range from 0 to 300 and is scored to reflect morphologically-relevant staining of cells.

### Statistical analysis

All statistical analyses were performed using the R statistical package. The H-scores from the two pathologists were averaged. Statistical differences between experimental conditions (A, B and clinical) were determined using a two-sided Wilcox Signed Rank test. P-values for these tests were then converted into false discovery rate corrected q-values because multiple comparisons were assessed simultaneously. Plots were generated using ggplot2.

## Data Availability

The datasets generated and analyzed during the current study are available from the corresponding author on reasonable request.

## References

[1] Agrawal, L., Engel, K. B., Greytak, S. R. & Moore, H. M. Understanding preanalytical variables and their effects on clinical biomarkers of oncology and immunotherapy. Semin *Cancer Biol*. **52**, 26–38 (2018 Oct 1). 10.1016/j.semcancer.2017.12.008 PMC6004232 29258857

[2] Bledsoe, J. R., Kamionek, M. & Mino-Kenudson, M. BRAF V600E immunohistochemistry is reliable in primary and metastatic colorectal carcinoma regardless of treatment status and shows high intratumoral homogeneity. *Am. J. Surg. Pathol*. **38**(10), 1418–28 (2014 Oct). 10.1097/PAS.0000000000000263 PMC4167743 24921639

[3] Routhier Caitlin Ann, Mochel Mark C., Lynch Kerry, Dias-Santagata Dora, Louis David N., Hoang Mai P. (2013). Comparison of 2 monoclonal antibodies for immunohistochemical detection of BRAF V600E mutation in malignant melanoma, pulmonary carcinoma, gastrointestinal carcinoma, thyroid carcinoma, and gliomas. Human Pathology.

[4] Chafin, D. *et al*. Rapid two-temperature formalin fixation. Koomen, J. M., editor. *PLoS One*. Jan **8**(1), e54138 (2013). 10.1371/journal.pone.0054138 PMC3548901 23349806

[5] Theiss, A. P., Chafin, D., Bauer, D. R., Grogan, T. M. & Baird, G. S. Immunohistochemistry of colorectal cancer biomarker phosphorylation requires controlled tissue fixation. Koomen, J. M., editor. *PLoS One*. **9**(11), e113608 (2014 Jan 19). 10.1371/journal.pone.0113608 PMC4237459 25409462

[6] Bussolati G, Annaratone L, Medico E, D’Armento G, Sapino A (2011). Formalin fixation at low temperature better preserves nucleic acid integrity. PLoS One..

[7] Bauer DR, Otter M, Chafin DR (2018). A New Paradigm for Tissue Diagnostics: Tools and Techniques to Standardize Tissue Collection, Transport, and Fixation. Curr. Pathobiol. Rep..

[8] Twomey, J. D., Brahme, N. N. & Zhang, B. Drug-biomarker co-development in oncology – 20 years and counting. *Drug. Resist Updat*. **30**, 48–62 (2017 Jan 1). 10.1016/j.drup.2017.02.002 28363335

[9] Janku, F., Yap, T. A. & Meric-Bernstam, F. Targeting the PI3K pathway in cancer: are we making headway? *Nat. Rev. Clin. Oncol*. **15**(5), 273–91 (2018 Mar 6). 10.1038/nrclinonc.2018.28 29508857

[10] Yang, J. *et al*. Targeting PI3K in cancer: mechanisms and advances in clinical trials. *Mol Cancer*. **18**(1), 26 (2019 Dec 19). 10.1186/s12943-019-0954-x PMC6379961 30782187

[11] Wolf Corinna, Jarutat Tiantom, Vega Harring Suzana, Haupt Kristin, Babitzki Galina, Bader Sabine, David Kerstin, Juhl Hartmut, Arbogast Susanne (2013). Determination of phosphorylated proteins in tissue specimens requires high-quality samples collected under stringent conditions. Histopathology.

[12] Neumeister V. M., Anagnostou V., Siddiqui S., England A. M., Zarrella E. R., Vassilakopoulou M., Parisi F., Kluger Y., Hicks D. G., Rimm D. L. (2012). Quantitative Assessment of Effect of Preanalytic Cold Ischemic Time on Protein Expression in Breast Cancer Tissues. JNCI Journal of the National Cancer Institute.

[13] Bonnas Christel, Specht Katja, Spleiss Olivia, Froehner Stefanie, Dietmann Gabriele, Krüger Juliane M., Arbogast Susanne, Feuerhake Friedrich (2012). Effects of cold ischemia and inflammatory tumor microenvironment on detection of PI3K/AKT and MAPK pathway activation patterns in clinical cancer samples. International Journal of Cancer.

[14] Pinhel, I. F. *et al*. Extreme loss of immunoreactive p-Akt and p-Erk1/2 during routine fixation of primary breast cancer. *Breast Cancer Res*. **12**(5), R76 (2010 Jan 28). 10.1186/bcr2719 PMC3096968 20920193

[15] Gündisch Sibylle, Annaratone Laura, Beese Christian, Drecol Enken, Marchiò Caterina, Quaglino Elena, Sapino Anna, Becker Karl-Friedrich, Bussolati Gianni (2015). Critical roles of specimen type and temperature before and during fixation in the detection of phosphoproteins in breast cancer tissues. Laboratory Investigation.

[16] Lerch, M. *et al*. Rapid tissue processing using a temperature-controlled collection device to preserve tumor biomarkers. *Cell Tissue Bank*., 10.1007/s10561-019-09800-8 (2019). 10.1007/s10561-019-09800-8 PMC7058599 31838727

[17] Lerch Melissa L., Bauer Daniel R., Chafin David, Theiss Abbey, Otter Michael, Baird Geoffrey S. (2017). Precision Medicine Starts With Preanalytics. Applied Immunohistochemistry & Molecular Morphology.

[18] Friemel J., Rechsteiner M., Frick L., Bohm F., Struckmann K., Egger M., Moch H., Heikenwalder M., Weber A. (2014). Intratumor Heterogeneity in Hepatocellular Carcinoma. Clinical Cancer Research.

[19] Norgan, A. P. *et al*. Radio-Frequency Identification Specimen Tracking to Improve Quality in Anatomic Pathology. *Arch Pathol Lab Med*., arpa.2019-0011-OA (2019 Jun 27). 10.5858/arpa.2019-0011-OA 31246113

[20] Nakhleh Raouf, Fitzgibbons Patrick L., Nowak Jan A., Najarian Robert M., Keren David F., Colgan Terence J., Colasacco Carol, Fatheree Lisa A. (2018). The Lifecycle of an Evidence-Based Laboratory Practice Guideline: Origin, Update, Affirmation, and Impact!. Archives of Pathology & Laboratory Medicine.

